# Twenty-Second Annual Report of the Managers of the New York State Lunatic Asylum, for the Year 1864

**Published:** 1865-05

**Authors:** 


Twenty-Second Annual Report of the Managers of the New York State Lunatic Asylum, for the year 1864.

This is a pamphlet of 32 pages, embracing the usual statements of facts in relation to finances, number of admissions, discharges, cures, deaths, &c., from one of the oldest and largest Lunatic Asylums in our country.

